# The 2024 diagnostic criteria for primary sclerosing cholangitis

**DOI:** 10.1007/s00535-025-02265-5

**Published:** 2025-06-12

**Authors:** Itaru Naitoh, Hiroyuki Isayama, Nobuhisa Akamatsu, Suguru Mizuno, Toshio Fujisawa, Nobuhiro Nakamoto, Yousuke Nakai, Shuichiro Umetsu, Mitsuyoshi Suzuki, Shintaro Yagi, Hironori Haga, Kenji Notohara, Katsuhiro Sano, Susumu Tazuma, Takahiro Nakazawa, Atsushi Tanaka

**Affiliations:** 1 Department of Gastroenterology, Nagoya City University Midori Municipal Hospital, Nagoya, Japan; 2 Department of Gastroenterology, Graduate School of Medicine, Juntendo University, 2-1-1 Hongo, Bunkyo City, Tokyo 113-8421 Japan; 3 Artificial Organ and Transplantation Surgery Division, Department of Surgery, Graduate School of Medicine, University of Tokyo, Tokyo, Japan; 4 Department of Gastroenterology and Hepatology, Faculty of Medicine, Saitama Medical University, Saitama, Japan; 5 Division of Gastroenterology and Hepatology, Department of Internal Medicine, Keio University School of Medicine, Tokyo, Japan; 6 Department of Internal Medicine, Institute of Gastroenterology, Tokyo Women’s Medical University, Tokyo, Japan; 7 Department of Pediatric Hepatology and Gastroenterology, Saiseikai Yokohamashi Tobu Hospital, Yokohama, Japan; 8 Department of Pediatrics, Juntendo University Faculty of Medicine, Tokyo, Japan; 9 Department of Hepato-Biliary-Pancreatic Surgery and Transplantation, Kanazawa University, Kanazawa, Japan; 10 Department of Diagnostic Pathology, Kyoto University Hospital, Kyoto, Japan; 11 Department of Anatomic Pathology, Kurashiki Central Hospital, Kurashiki, Okayama Japan; 12 Department of Radiology, Juntendo University Graduate School of Medicine, Tokyo, Japan; 13 JR Hiroshima Hospital, Hiroshima, Japan; 14 Department of Gastroenterology and Metabolism, Nagoya City University Graduate School of Medical Sciences, Nagoya, Japan; 15 Department of Medicine, Teikyo University School of Medicine, Tokyo, Japan

**Keywords:** Diagnostic criteria, Primary sclerosing cholangitis, Large duct primary sclerosing cholangitis, Small duct primary sclerosing cholangitis, Recurrence following liver transplantation

## Abstract

Primary sclerosing cholangitis (PSC) is an idiopathic chronic cholestatic disease with a poor prognosis. As there were no specific biomarkers for diagnosing PSC, we developed diagnostic criteria in 2016 based on cholangiography and elevated biliary enzymes. Novel findings and knowledge have subsequently accumulated, and we now propose the 2024 diagnostic criteria, to overcome several limitations of the 2016 diagnostic criteria. The Intractable Hepato-Biliary Diseases Study Group in Japan of the Committee of Research on Measures for Intractable Diseases established a working group consisting of experts in PSC comprising gastroenterologists, endoscopists, hepatologists, liver-transplant surgeons, pediatric hepatologists, pathologists, and radiologists. This working group proposed the 2024 diagnostic criteria after several discussions and public hearings. There are additional diagnostic targets; small duct PSC, pediatric PSC, and PSC recurrence following liver transplantation differ from the 2016 diagnostic criteria, which were for diagnosing large duct PSC in adults. The 2024 diagnostic criteria facilitate the use of magnetic resonance cholangiography in addition to endoscopic retrograde cholangiography in imaging, and incorporate gamma-glutamyl transferase for evaluating cholestasis to diagnose pediatric patients. Furthermore, PSC recurrence following liver transplantation can be diagnosed based on a liver biopsy and characteristic biliary findings. We hope that the 2024 diagnostic criteria will help not only hepatologists treating adults but also general physicians, pediatric hepatologists, and liver-transplant surgeons who manage patients with various forms of PSC.

## Introduction

Primary sclerosing cholangitis (PSC) is an idiopathic chronic cholestatic disease that is caused by multifocal fibrous strictures of the intra- and extra-hepatic bile ducts [1, 2]. The etiology and pathophysiology of PSC remain unclear. The majority of PSC cases have biliary strictures on cholangiography, and this type of PSC is called large duct PSC. By contrast, small duct PSC is characterized by normal cholangiography and histological features of PSC on liver biopsy. Inflammatory bowel disease (IBD) is frequently associated with PSC. The natural history of PSC exhibits considerable variability among individuals, ranging from minimal progression to rapid deterioration, often with an unpredictable trajectory. In general, the disease follows a gradual course, characterized by alternating periods of improvement and exacerbation in blood test results and clinical symptoms, frequently associated with episodes of cholangitis. Despite medical treatment, the biliary strictures frequently result in fluctuating cholestasis, hepatic cirrhosis, and liver failure. PSC has an increased risk of developing hepatobiliary malignancies, especially cholangiocarcinoma (CCA), while colorectal cancer is associated with concurrent IBD [3]. The prognosis of PSC is poor, and the major causes of death are CCA, liver failure, and variceal bleeding [4]. In Japan, the 5-year and 10-year survival rates are 81.3% and 69.9%, respectively, and the 5-year and 10-year survival rates without liver transplantation (LTx) are 77.4% and 54.9%, respectively [5]. Although ursodeoxycholic acid (UDCA) has been associated with an improved long-term outcome in Japanese patients with PSC [6], its effectiveness is limited and liver transplantation is the only potentially curative treatment.

No biomarkers with high sensitivity and specificity are available for diagnosing PSC, and the diagnostic criteria for PSC proposed by the Mayo Clinic have long been used internationally for diagnosing PSC [7]. We proposed the 2016 diagnostic criteria for PSC in Japan [8] because a Japanese nationwide survey and epidemiological studies revealed that several clinical aspects of PSC differed between Japan and Western countries [5, 9–11]. In terms of epidemiology, we conducted a nationwide epidemiological survey in 2016 and found that the prevalence of patients with PSC in Japan was 1.80 (95% CI 1.75–1.85) per 100,000 population, indicating a roughly twofold increase over the past 9 years. [11]. It is of note that the prevalence of PSC in Japan is considerably lower than in the West. To further delineate the present status of PSC in Japan, we conducted a nationwide survey of PSC in 2015 with the cooperation of the Japan Biliary Association and reported on the characteristics of 435 cases accumulated [5]. The male-to-female ratio and median age at diagnosis were found to be consistent with those observed in Europe and the United States. On the other hand, an analysis of the age distribution at diagnosis revealed a peak not only in younger patients but also in older patients, and the latter was not observed in most previous Western studies. The rate of IBD comorbidity was 40%, which was lower than in Europe and the United States. These observations, which were also noted in the previous nationwide survey conducted in 2013 [9], were identified as clinical characteristics unique to Japan.

Subsequently, novel findings and knowledge have accumulated, and several clinical practice guidelines for PSC, including the Japanese one, have been published [12–14]. Therefore, the Intractable Hepato-Biliary Diseases Study Group in Japan (Chairperson, Atsushi Tanaka) of the Committee of Research on Measures for Intractable Diseases established a working group (Chairperson, Hiroyuki Isayama) of experts in PSC to revise the 2016 diagnostic criteria for PSC.

## Organization and process of the revising

Revised criteria that included small duct PSC, PSC in pediatric patients, and PSC recurrence following LTx were required. Therefore, the working group included gastroenterologists, endoscopists, hepatologists, liver-transplant surgeons, pediatric hepatologists, pathologists, and radiologists. The committee members drafted the new criteria after several face-to-face and online meetings, in addition to intermittent e-communications. The evaluating committee members checked the draft and made comments for creating committee members. The draft was presented at a meeting of the Intractable Hepato-Biliary Diseases Study Group in Japan and sent to the members. After some revision, we held public hearings from members of the Japanese Society of Gastroenterology on websites. After the public hearings and frequent e-communications, the revised version was confirmed, and the final version of the 2024 diagnostic criteria was proposed.

## Concept of revision

The 2024 diagnostic criteria follow the basic concepts of the 2016 diagnostic criteria for PSC. The 2016 diagnostic criteria were designed for diagnosing only large duct PSC. In comparison, the 2024 diagnostic criteria are also designed for diagnosing small duct PSC and PSC recurrence following LTx because of the recent increases in these conditions. Regarding imaging, only the cholangiographic findings were incorporated in the 2016 diagnostic criteria for evaluating biliary findings, and endoscopic retrograde cholangiography (ERC) has conventionally been used as the gold standard for evaluating cholangiographic findings. However, magnetic resonance cholangiography (MRC) has become the standard procedure because of recent improvements in image quality and because it is less invasive. Therefore, the 2024 diagnostic criteria use MRC as the first diagnostic modality and incorporate other modalities in addition to cholangiography because new knowledge of biliary findings in PSC has accumulated. Colonoscopy has not been conducted sufficiently for evaluating IBD in patients with PSC according to previous Japanese national surveys, although IBD is crucial for diagnosing PSC. We recommend colonoscopy in the diagnosis for PSC by prioritizing the existence of IBD in the 2024 diagnostic criteria. In the laboratory evaluation of cholestasis, only alkaline phosphatase (ALP) was used as a biliary enzyme in the 2016 diagnostic criteria. In clinical practice, however, it is difficult to diagnose pediatric PSC because ALP is often inappropriate as a biliary enzyme for evaluating cholestasis in pediatric patients. Therefore, the 2024 diagnostic criteria add gamma-glutamyl transpeptidase (GGT) as another biliary enzyme for diagnosing pediatric PSC patients.

## Small duct PSC

Large duct PSC shows biliary strictures on cholangiography, and comprises the majority of PSC. By contrast, small duct PSC exhibits normal cholangiographic results but displays histological findings consistent with PSC [15]. Small duct PSC is much less prevalent, and its natural history and long-term outcomes of small duct PSC have not been clarified. It is still unclear whether small duct PSC is an early stage of large duct PSC, or whether small and large duct PSC are two distinct entities. Two recent studies reported progression to large duct PSC in 33% and 55% of small duct PSC during follow-up [16, 17]. Consequently, some small duct PSC progresses to large duct PSC. In this regard, it is imperative to accurately diagnose small duct PSC and meticulously monitor its progression.

The diagnosis of small duct PSC is made on the basis of histological findings that are consistent with PSC in the context of a normal cholangiography. Among the diagnostic criteria proposed for PSC, the diagnostic criteria established by the International PSC Study Group can be used in the diagnosis of small duct PSC [18]. However, small duct PSC cannot be diagnosed using PSC2016 now in Japan, and as a result, the number of patients with small duct PSC is limited and the data available on small duct PSC is scarce in Japan.

## Pediatric PSC

The clinical characteristics of pediatric PSC are similar to those of adult PSC. The respective incidence of IBD and of small duct PSC were 76% and 13% of pediatric PSC patients in an international multicenter study [19], and 33% and 8% in a Japanese single-center study [20]. Dominant strictures, cholangitis, and CCA are less common with pediatric PSC than with adult PSC, and pediatric PSC appears milder than adult PSC. The 5-year LTx-free survival is 88% in pediatric patients [19], and is higher than in adult PSC. PSC-autoimmune hepatitis (AIH) overlap (PSC-AIH) is observed in 33% of pediatric PSC [19], which is higher than in adult PSC. However, there is no evidence that pediatric patients have a distinct phenotype of PSC.

## The 2024 diagnostic criteria for PSC

**Fig. 1:**
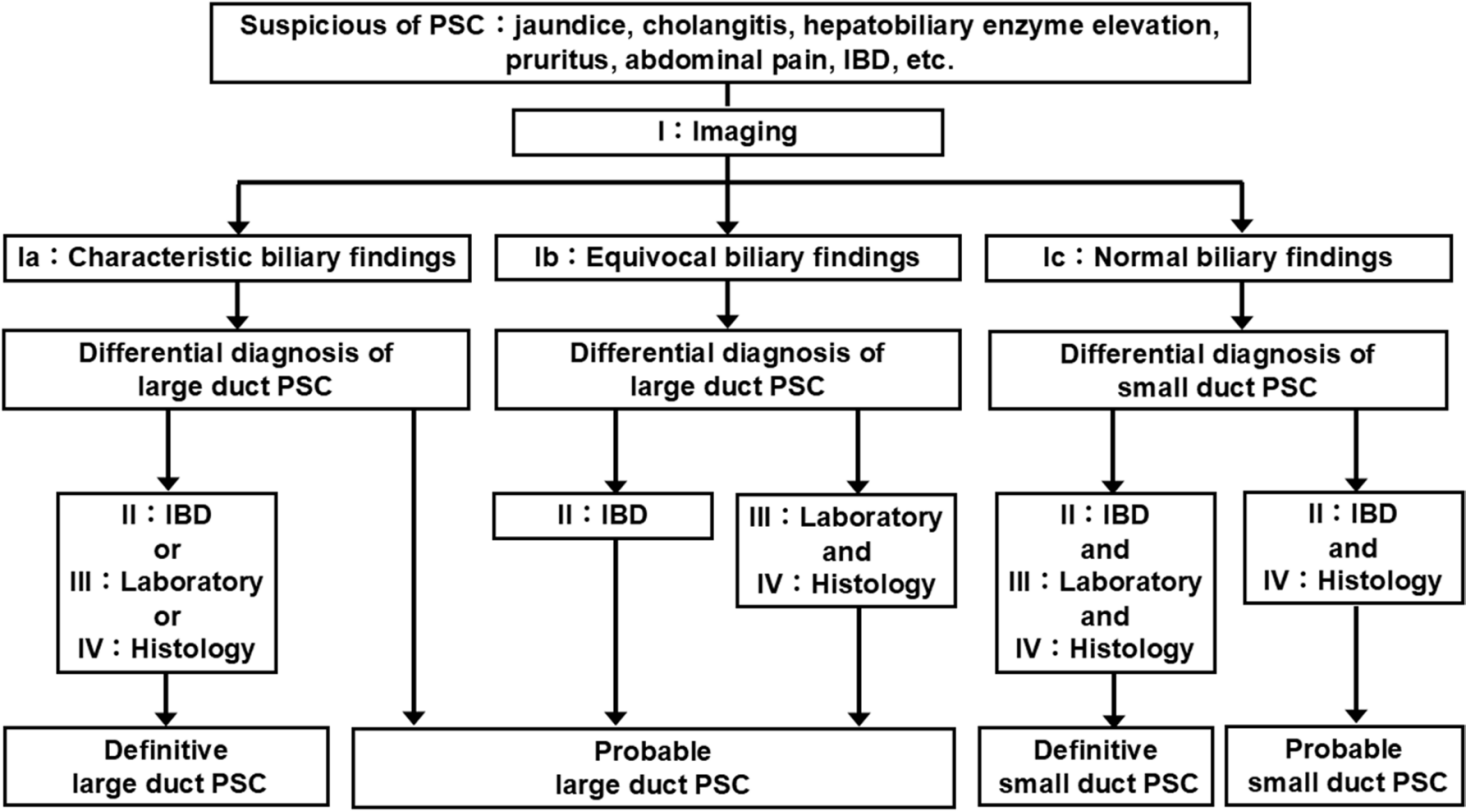
Flowchart of the 2024 diagnostic criteria for primary sclerosing cholangitis

The working group proposed the final version of the 2024 diagnostic criteria for PSC (Table 1). PSC can be diagnosed after differentiating IgG4-related sclerosing cholangitis (IgG4-SC), secondary sclerosing cholangitis, malignant diseases, and liver diseases. The differential diagnoses of large and small duct PSC are shown in Table 2. Definitive or probable cases are diagnosed as PSC. Figure 1 shows the flowchart of the 2024 diagnostic criteria for PSC.

**Table 1:** The 2024 diagnostic criteria for primary sclerosing cholangitis

I: Imaging
Ia: Characteristic biliary findings (one of the followings)
1)Multifocal band-like strictures
2)Beaded appearance
3)Pruned-tree appearance
4)Diverticulum-like outpouching
Ib: Equivocal biliary findings
Ic: Normal biliary findings
II: Concurrent inflammatory bowel disease
III: Laboratory
Elevated ALP level in adult/GGT level in children (under 16 years old)
IV: Histology
Onion-skin lesion/fibrous obliterative cholangitis in liver biopsy
PSC can be diagnosed after differentiating IgG4-related sclerosing cholangitis, secondary sclerosing cholangitis, malignant disease, and liver disease
Differential diagnosis of PSC is shown in Table 2
Diagnosis of large duct PSC
Definitive: Ia + II/III/IV
Probable: Ia, Ib + II, Ib + III + IV
Possible: Ib + III/IV
Diagnosis of small duct PSC
Definitive: Ic + II + III + IV
Probable: Ic + II + IV
Possible: Ic + II/IV + III

“ + ” refers to “and”, and “/” refers to “or”

**Table 2:** Differential diagnosis of primary sclerosing cholangitis

Type	Etiology	Disease
Large duct PSC	Anatomic	Choledocholithiasis
		Chronic pancreatitis
		Surgical biliary trauma
		Anastomotic stricture
		Radiation injury
	Genetic	Biliary atresia
		CD40 ligand deficiency (hyper-IgM syndrome)
		DCDC2 mutations
	Immunological	IgG4-related sclerosing cholangitis (IgG4-SC)
		Eosinophilic cholangitis
		Mast cell cholangiopathy
		Immune checkpoint inhibitor (ICI)-induced cholangitis
		Sarcoidosis
		Hepatic allograft rejection (including T-cell mediated, chronic, and antibody-mediated rejection)
	Infectious	Recurrent pyogenic cholangitis
		Parasitic cholangiopathy (cryptosporidiosis, microsporidiosis, liver fluke, ascariasis)
		Cytomegalovirus
		Human immunodeficiency virus (HIV)-associated cholangiopathy
	Ischemic	Critically ill patients (including post-COVID)
		Systemic vasculitis
		Hepatic artery thrombosis
		Vascular trauma
		Intra-arterial chemotherapy
	Malignant	Cholangiocarcinoma
		Langerhans cell histiocytosis (Histiocytosis X)
		Hodgkin’s disease
Small duct PSC	Genetic	Alagille syndrome
		Cystic fibrosis
		ABCB4 deficiency (progressive familial intrahepatic cholestasis)
	Immunological	Primary biliary cholangitis (PBC)
		Drug-induced liver injury (DILI)
		Graft-versus-host disease (GVHD)
		Hepatic allograft rejection (including T-cell-mediated, chronic, and antibody-mediated rejection)
	Infectious	Sepsis causing cholestasis
	Metabolic	Alcohol-associated liver disease (ALD)
		Metabolic dysfunction associated fatty liver disease (MAFLD)

## Imaging

**Fig. 2:**
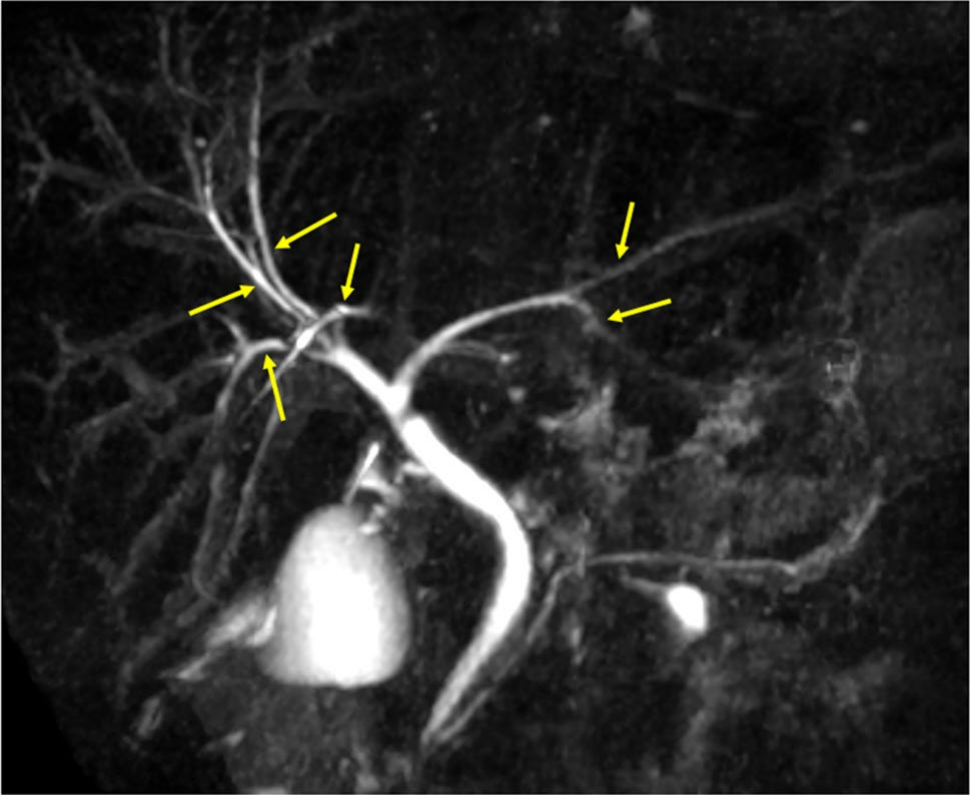
Maximum intensity projection image of a normal biliary tree. Arrows: tertiary (sub-segmental) branches

Cholangiographic findings are crucial in the diagnosis of large duct PSC and for distinguishing between small and large duct PSC. MRC and ERC can both be used for evaluating the cholangiographic findings. The diagnostic accuracy of MRC is comparable to that of ERC in the diagnosis of PSC [21]. A meta-analysis of the diagnostic performance of MRC revealed high sensitivity (86%) and specificity (94%) for diagnosing PSC [22]. Therefore, MRC is recommended as the first diagnostic modality in the 2024 diagnostic criteria because of the recent improvement in image quality and its less-invasive nature. We also propose MRC protocol and image quality guidelines to standardize them for evaluating the cholangiographic findings of PSC (Table 3, Fig. 2). In addition to ERC and MRC, computed tomography (CT), MRI, endoscopic ultrasonography (EUS), intraductal ultrasonography (IDUS), and peroral cholangioscopy (POCS) are incorporated as modalities for evaluating the biliary findings.

**Table 3:** Magnetic resonance cholangiography protocol and image quality guidelines

The following are mandatory for evaluating the cholangiographic findings on magnetic resonance cholangiography (MRC). Endoscopic retrograde cholangiography (ERC) should be considered when MRC with the following qualities is not obtained
1. At least 1.5 Tesla field strength
2. Secondary (segmental) branches seen on maximum intensity projection (MIP) images of three-dimensional (3D) MRC sequences. Effort should be made to visualize tertiary (sub-segmental) branches and more peripheral bile ducts
3. Two-dimensional (2D) MRC sequence imaging in multiple directions when secondary branches are not seen in 3D MRC sequence MIP images

**Fig. 3:**
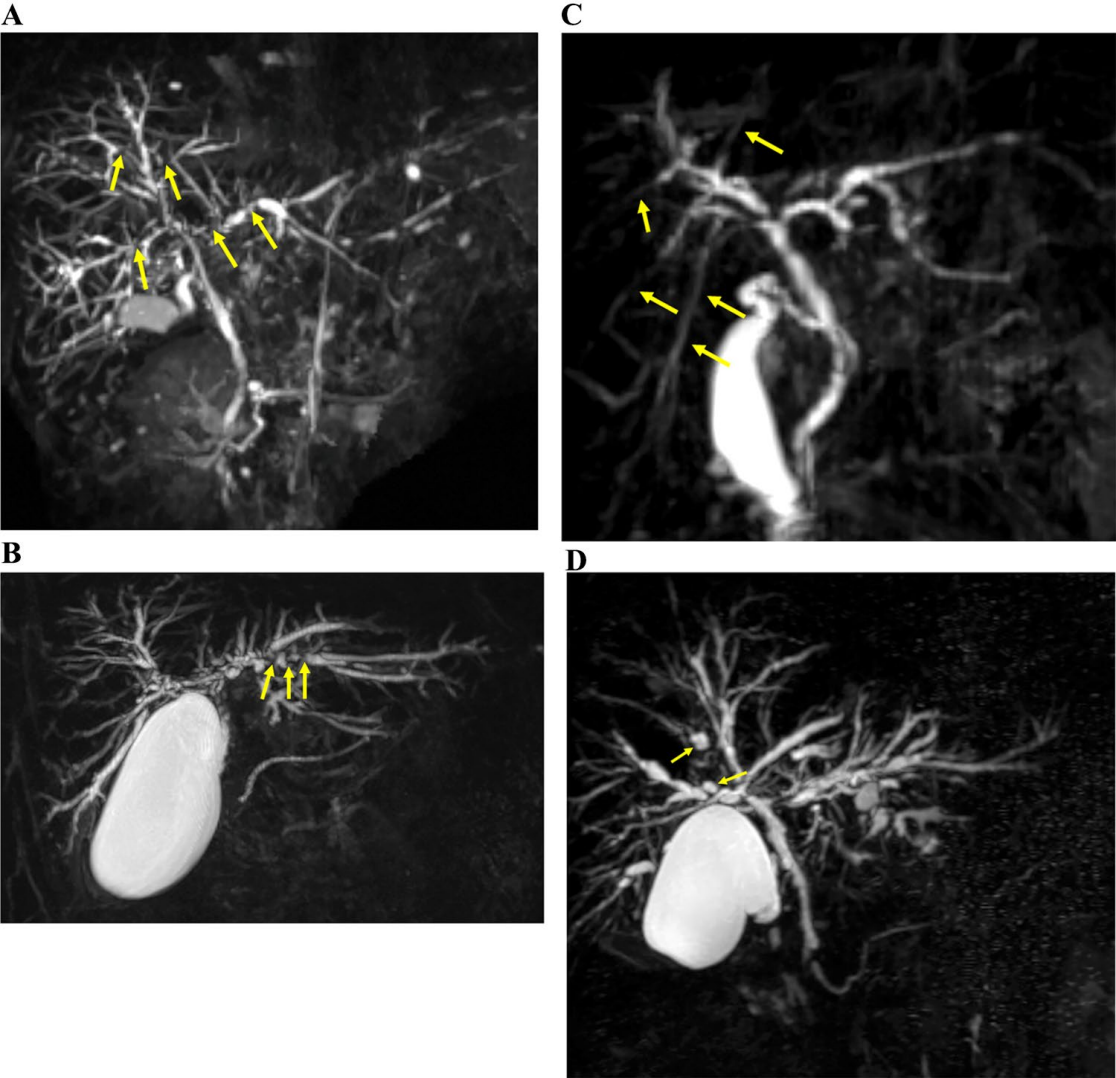
Characteristic biliary findings on magnetic resonance cholangiography. **A** Multifocal band-like strictures (arrows) are multiple short strictures (1–2 mm). **B** Beaded appearance (arrows), a short, annular stricture alternating with normal or minimally dilated segments. **C** Pruned-tree appearance (arrows), defined as a narrowing of the intrahepatic duct, and reduced arborization of the intrahepatic duct and pruning. **D** Diverticulum-like outpouching (arrows) is outpouching resembling diverticula, often protruding between adjacent strictures

**Fig. 4:**
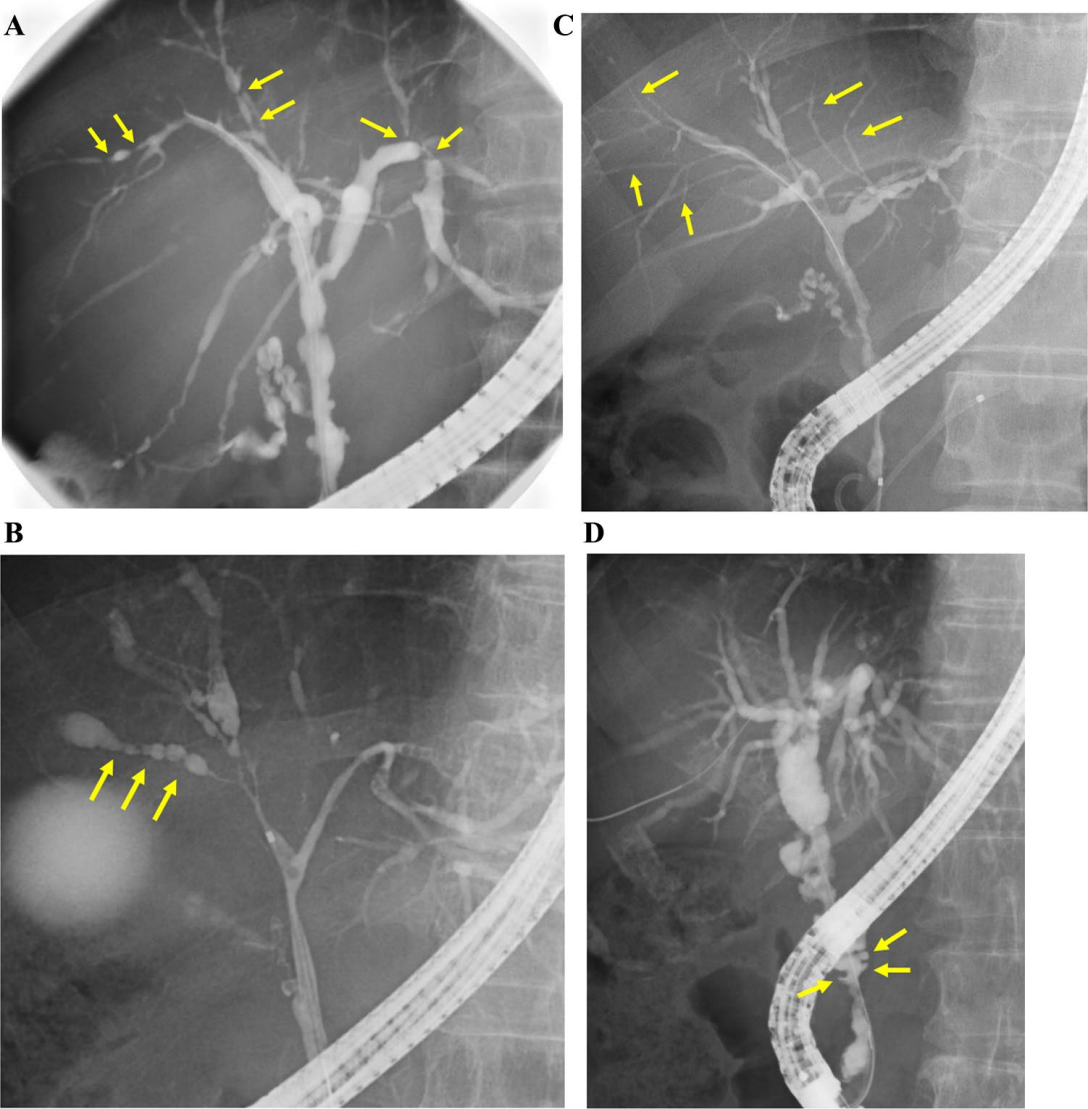
Characteristic biliary findings on endoscopic retrograde cholangiography. **A** Multifocal band-like strictures (arrows). **B** Beaded appearance (arrows). **C** Pruned-tree appearance (arrows). **D** Diverticulum-like outpouching (arrows)

Multifocal band-like strictures, a beaded appearance, a pruned-tree appearance, and diverticulum-like outpouching have been described as characteristic biliary findings on cholangiography [23]. We defined these four findings as characteristic biliary findings. Imaging meets characteristic biliary findings when any of these four characteristic biliary findings are observed. The four characteristic biliary findings on MRC and ERC are shown in Figs. 3 and 4, respectively. Multifocal band-like structures are multiple short strictures (1–2 mm) (Figs. 3A and 4A). Short strictures are characteristic of PSC, while segmental or long strictures (> 3 mm) are characteristic of IgG4-SC. Single band-like strictures should be differentiated from CCA. A beaded appearance is a short annular stricture alternating with normal or minimally dilated segments (Figs. 3B and 4B). A pruned-tree appearance is defined as narrowing of the intrahepatic duct, and reduced arborization of the intrahepatic duct and pruning (Figs. 3C and 4C). Diverticulum-like outpouching is outpouching that resembles diverticula, often protruding between adjacent strictures (Figs. 3D and 4D). Diverticulum-like outpouching observed on IDUS or POCS meets the characteristic biliary findings even if it is not seen on cholangiography.

Equivocal biliary findings are defined as biliary strictures or sclerosing findings that do not meet the above four characteristic biliary findings. Diffuse thickening of the bile duct wall is an equivocal biliary finding suggestive of early PSC. Typical IDUS findings of PSC are circular-asymmetrical wall thickening, an irregular inner margin, and disappearance of the three layers [24]; these findings are also observed on EUS. Focal thickening of the bile duct wall should be differentiated from CCA. Longitudinal and multiple ulcer scars on POCS are an equivocal biliary finding. Normal biliary findings are incorporated for diagnosing small duct PSC.

## Concurrent inflammatory bowel disease

Primary sclerosing cholangitis is closely associated with IBD. Concurrent IBD occurs in 60–80% of patients with PSC in western countries [3], whereas in Japan, the comorbidity rate is approximately 40% based on a 2015 national survey [5]. Most cases of concurrent IBD involve ulcerative colitis (UC), while Crohn’s disease is less common (comorbid rate approximately 20%). In addition, non-specific IBD characterized by right-side colitis, backwash ileitis, and rectal sparing is frequently observed in patients with PSC [25]. Concurrent IBD is an important clue for diagnosing PSC. Intestinal lesions associated with PSC are often mild or asymptomatic, and further investigation is necessary to determine the causal association with the clinical course and prognosis of PSC. Therefore, we recommend routine colonoscopy in the 2024 diagnostic criteria, even if gastrointestinal symptoms are not observed. The role of gut microbiota in the pathogenesis of PSC has recently gained attention, and certain intestinal bacteria, including *Klebsiella pneumoniae*, are associated with the course and prognosis in Japanese and European cohorts [26, 27].

## Laboratory

Cholestasis is a characteristic, but non-specific, laboratory finding of PSC. Alkaline phosphatase is used as a biliary enzyme for evaluating cholestasis. A Japanese nationwide survey in 2015 revealed that the average serum ALP levels were 2.2 × the upper limit of normal [5]. However, ALP is not appropriate for evaluating cholestasis in pediatric patients because bone turnover and growth increase serum ALP levels. Therefore, GGT is recommended as a biliary enzyme in patients under 16 years old in the 2024 diagnostic criteria. There are no laboratory diagnostic markers specific for PSC. Recently, anti-integrin αvβ6 autoantibody has been suggested as a diagnostic laboratory marker for PSC; it has a sensitivity of 50% in PSC patients without UC, and a sensitivity of 95% and specificity of 94% in patients with UC [28]. However, anti-integrin αvβ6 autoantibody has not yet been approved for use in daily clinical practice under the Japanese health insurance system.

## Histology

**Fig. 5:**
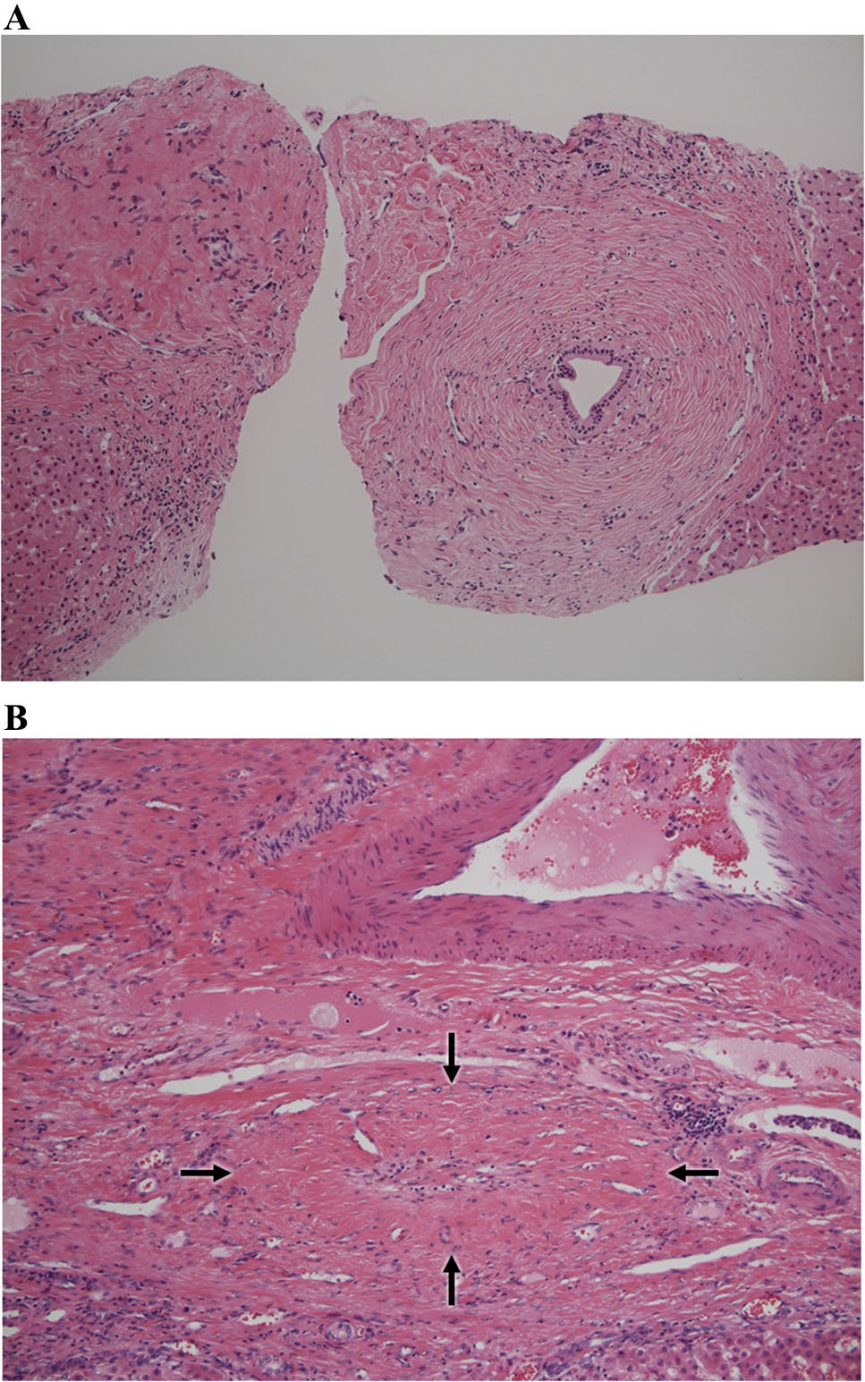
Histology of the liver biopsy. **A** Onion-skin lesion (hematoxylin and eosin staining), concentric fibrosis around the bile duct epithelium. **B** Fibrous obliterative cholangitis (arrows, hematoxylin and eosin staining), fibrous occlusion of the bile duct lumen due to the progression of an onion-skin lesion

An onion-skin lesion is concentric fibrosis around the bile duct epithelium (Fig. 5A) [29]. In the 2024 diagnostic criteria, onion-skin lesions are limited to those associated with degeneration or the disappearance of bile duct epithelium. Fibrous obliterative cholangitis is fibrous occlusion of the bile duct lumen caused by the progression of an onion-skin lesion (Fig. 5B) [30]. Both onion-skin lesions and fibrous obliterative cholangitis are characteristic, but not specific, findings of PSC in liver biopsies, as similar findings are seen in hepatolithiasis [31]. In addition, the rate of occurrence of these findings is low (7–50%) in PSC liver biopsies [13]. Therefore, a liver biopsy is not required in patients who can be diagnosed based on other clinical findings, but it is mandatory in the diagnosis of small duct PSC or PSC-AIH overlap syndrome, as well as for excluding other biliary diseases that mimic PSC.

## PSC recurrence following liver transplantation

Recurrent PSC among recipients who underwent LTx for PSC definitely impairs the graft and patient survival after LTx, which results in poor outcomes [32]. The incidence of recurrent PSC varies widely among transplant centers, which may reflect differences in the diagnostic criteria, length and type of follow-up, and inclusion of protocol biopsies. Reported recurrence rates average 17.7 to 18.5%, with an approximately 50% incidence of graft loss among recurrent recipients [33, 34]. Risk factors associated with PSC recurrence include CCA before LTx, co-existing active IBD, older donor age, a higher MELD score, episode of acute cellular rejection, and biliary anastomotic complications, while colectomy before LTx and good control of IBD activity were protective. Consistent with other autoimmune liver diseases, disease recurrence is common after LTx for PSC. Consequently, differentiating recurrent PSC, rejection, and secondary non-anastomotic stricture is of the utmost importance for prompt appropriate treatment.

## The diagnostic criteria for PSC recurrence following liver transplantation

The working group established their final proposal for the diagnostic criteria for PSC recurrence following LTx as a part of the 2024 diagnostic criteria (Table 4). PSC recurrence following LTx can be diagnosed after differentiating several diseases. Caution is necessary when considering recurrent PSC in patients who develop a non-anastomotic biliary stricture within 90 days after LTx. Definitive or probable cases are diagnosed as PSC recurrence following LTx.

**Table 4:** Diagnostic criteria for primary sclerosing cholangitis recurrence following liver transplantation

I: Confirmed diagnosis of PSC prior to liver transplantation
II: Histology
Onion-skin lesion/fibrous obliterative cholangitis in liver biopsy
III: Imaging > 90 days post-liver transplantation
IIIa: Characteristic biliary findings (one of the followings)
1)Multifocal band-like strictures
2)Beaded appearance
3)Pruned-tree appearance
4)Diverticulum-like outpouching
IIIb: Equivocal biliary findings
Diagnosis of PSC recurrence following liver transplantation
Definitive: I + II + III
Probable: I + II/IIIa
Possible: I + IIIb
A diagnosis of PSC recurrence following LTx can be made after differentiating the following. Recurrent PSC should be considered in patients developing a non-anastomotic biliary stricture within 90 days after LTx without the following factors
A) Non-anastomotic biliary stricture following hepatic arterial thrombosis
B) Ductopenia or non-anastomotic biliary stricture due to rejection proven by liver biopsy
C) Secondary non-anastomotic biliary stricture following biliary anastomotic complications (biliary leakage and stricture) *
D) Non-anastomotic biliary stricture due to antibody-mediated rejection (especially in ABO blood type incompatible transplantation)

^*^Recurrent PSC often develops after an anastomotic biliary stricture

“ + ” refers to “and”, and “/” refers to “or”

## Histology of PSC recurrence following liver transplantation

The histological findings in a liver biopsy are crucial for diagnosing recurrent PSC in LTx recipients. Onion-skin lesion and fibrous obliterative cholangitis in a liver biopsy are the most important findings for the histological diagnosis of recurrent PSC. For the early diagnosis of recurrence, it is important to detect early histological changes, such as mild portal edema, sinusoidal neutrophils, and mild hepatocanalicular cholestasis [35]. More importantly, when diagnosing recurrent PSC, it is important to differentiate rejection, de novo AIH, and secondary sclerosing cholangitis due to rejection or biliary anastomotic complications, which requires a histological diagnosis by an experienced pathologist.

## Imaging for PSC recurrence following liver transplantation

The cholangiographic findings of PSC recurrence following LTx vary. Characteristic and equivocal biliary findings are observed in PSC recurrence, and the definitions of these findings are the same as given above in the image section. Histological examination is required when equivocal biliary findings are observed. Biliary findings should be evaluated 90 days after LTx. MRC and direct cholangiography such as ERC or percutaneous transhepatic cholangiography can be used to evaluate the biliary findings. The MRC protocol and image quality guidelines for evaluating cholangiography are the same as given above in the image section (Table 3).

## Conclusion

We have proposed the 2024 diagnostic criteria for PSC, to overcome several limitations of the 2016 diagnostic criteria. We hope that the 2024 diagnostic criteria will help not only hepatologists dealing with adult patients but also general physicians, pediatric hepatologists, and liver-transplant surgeons who manage PSC patients with various conditions.

## Abbreviations

AIH: Autoimmune hepatitis
ALP: Alkaline phosphatase
CCA: Cholangiocarcinoma
ERC: Endoscopic retrograde cholangiography
EUS: Endoscopic ultrasonography
GGT: Gamma-glutamyl transpeptidase
IBD: Inflammatory bowel disease
IDUS: Intraductal ultrasonography
IgG4-SC: IgG4-related sclerosing cholangitis
LTx: Liver transplantation
MRC: Magnetic resonance cholangiography
POCS: Peroral cholangioscopy
PSC: Primary sclerosing cholangitis
UDCA: Ursodeoxycholic acid
